# Mini review: Bidirectional Regulation of Circadian Rhythm by Suprachiasmatic Nucleus and Nuclear Receptors in Female Mammals

**DOI:** 10.5334/jcr.245

**Published:** 2025-04-08

**Authors:** Dharani Abirama Sundari Shanmugam, Ashwini Devi Balaraman, Abhijit Kar, Abishek Franco, B. Arjun Chandra Balaji, S. Meenakumari, P. K. Praveenkumar, R. Gayathri, Vinoth Kumar Ganesan, Merugumolu Vijay Kumar, K. Senthilkumar, B. Shanthi

**Affiliations:** 1 Department of Endocrinology, Dr. ALM. PG. Institute of Basic Medical Sciences, University of Madras, Taramani, Chennai – 600113, Tamil Nadu, India; 2 Department of Biotechnology, School of Bioengineering, SRM Institute of Science and Technology, Kattankulathur – 603202, Tamil Nadu, India; 3 Department of Computer Science, Dalhousie University, Halifax, Nova Scotia, B3H 4R2, Canada; 4 Department of Biotechnology, Sri Venkateswara College of Engineering, Sriperumbudur Tk – 602117, Tamil Nadu, India; 5 Department of Biotechnology, St Joseph’s College of Engineering, Old Mahabalipuram Road, Kamaraj Nagar, Semmancheri, Chennai – 600119, Tamil Nadu, India; 6 Department of Health Research (DHR-ICMR), Multi-Disciplinary Research Unit (MRU), Rangaraya Medical College, Kakinada – 533003, Andhra Pradesh, India; 7 Department of Pharmacology, Dayananda Sagar University, Bengaluru – 560078, Karnataka, India; 8 Mr. & Mrs. Ko Chi-Ming Centre for Parkinson’s Disease Research, School of Chinese Medicine, Hong Kong Baptist University, Kowloon Tong, Hong Kong Special Administrative Region of China; 9 Department of Biotechnology, JAASB Institute and Research Academia, Valasaravakkam, Chennai – 600087, Tamil Nadu, India

**Keywords:** Circadian rhythm, suprachiasmatic nucleus (SCN), CLOCK genes, nuclear receptors

## Abstract

The anterior region of the hypothalamus accommodates a bilateral structure called the suprachiasmatic nucleus (SCN), which controls, modulates, and perpetuates the homeostasis of circadian rhythm and sleep hormone release. These SCN have a predominance over multitudinous peripheral tissues like the uterus, liver, intestine, pancreas, endocrine system, immune system, reproductive system, and cardiovascular system. This peripheral clock acts as a pacemaker for circadian rhythm timing, which regulates crucial metabolic pathways and organizes numerous activities in the female reproductive network of mammals. The circadian *CLOCK* genes are expressed in various reproductive organs. The *CLOCK, BMAL1, CRY*, and *PER* genes harmonize the balance and manifestation of nuclear receptors (NRs) expression, and the other way round, NRs regulate these circadian genes. Several NRs, in particular estrogen, progesterone, androgen, and PPARs, nurture the ovary and uterus. Bidirectional coordination between SCN and NRs maintains the circadian rhythm of the hypothalamic-pituitary-gonadal (HPG) axis of the female reproductive organs.

## 1. Graphical abstract

**Figure:**
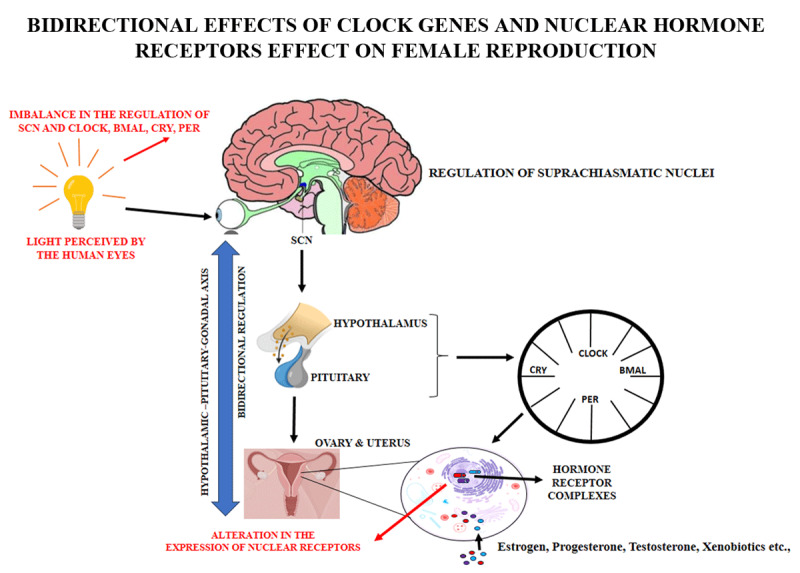
(Images were procured from the sources: www.biorender.com and Enrique A Gil, 2017 [141] and then subsequently modified).

## 2. Objective and Methodology

The conceptual studies on the perception of the circadian rhythm and its cognizance on the hypothalamic-pituitary-gonadal (HPG) axis still need a progressing interpretation. Innumerable studies have investigated the bidirectional coordination and harmonization between the suprachiasmatic nucleus and nuclear receptors in maintaining the circadian rhythm. Their interdependence in the regulation of circadian rhythm by nuclear receptors and vice versa still needs more adequate understanding of how the suprachiasmatic nucleus, *CLOCK* genes, and HPG axis were systematized by nuclear receptors. Numerous complex diseases and their pathogenesis in the female reproductive system were associated with poor coordination of circadian rhythm and the HPG axis, but it still prevails to ascertain the methodology for deciphering how the regulation and homeostasis of circadian rhythm by nuclear receptors transpire. This is the prime objective for the commencement of our mini-review. The methodology of data sourcing and intervention was entirely based on online resources like PubMed, MEDLINE, SCOPUS, EBSCO, SCINDEX, Wiley, Embase, and Google Scholar. The main keywords for the online search approach strategy comprise circadian rhythm, suprachiasmatic nucleus (SCN), HPG axis, female reproductive system, *CLOCK* genes, nuclear receptors, estrogen, progesterone, androgen, and PPAR. We have analyzed several review and research articles, short communications, case studies on humans, rats, and other mammals, and suitable articles with current investigations and up-to-date coverage that were appropriate were chosen for our contemporary work. The conclusion and evaluation of our mini review were further discussed.

## 3. Circadian rhythm: Introduction

Circadian rhythms in humans include a varied range of physiological and behavioral aspects like short-term memory, sleep propensity, core body temperature, urine production, hormones, subjective alertness, cognitive performance, and sleep structure [1,2]. These rhythms are synchronized to the 24-hour solar day in normal conditions, and with the light-dark/activity-rest cycle, they show specific phase relationships with each other. A central circadian pacemaker is located in the suprachiasmatic nucleus (SCN) of the hypothalamus, which orchestrates multiple circadian oscillators in the periphery drives of the circadian set in mammals [3]. Classical photoreceptors (rods and cones), as well as a more recently identified subset of intrinsically-photosensitive retinal ganglion cells, are the light input for SCN [4,5]. An acute shift in the light-dark cycle can alter the phase of the circadian rhythms [6]. Bidirectional communication exists between the circadian and reproductive systems through the brain and periphery to regulate the timing of essential reproductive events. The circadian clock genes are rhythmically expressed in the female reproductive organs, including the ovary, fallopian tube (oviduct or uterine tube in rodents), and uterus. In the suprachiasmatic nucleus (SCN) of the hypothalamus, the central circadian clock is found, and it generates daily oscillations [7], which contribute to organizing the timing of most behaviors and physiological events in mammals, especially the luteinizing hormone (LH), which surges during the estrous cycle that triggers ovulation, and also releases and revives the follicle stimulating hormone (FSH) [8,9]. The LH surge occurs on the day of proestrus at a specific “critical period” in the late afternoon, approximately 2–4 hours before lights off in the cycling female rodents [10,11]. SCN-lesions in female rats can abolish the LH surge and prevent ovulation [11].

### 3.1 Circadian rhythm: hypothalamic-pituitary-gonadal (HPG) axis

Circadian rhythm enact a significant role in maintaining the hypothalamic-pituitary-gonadal (HPG) axis and the release of gonadotropin-releasing hormone (GnRH) in diverse female mammals [9]. Reproductive events, in turn, have both direct and indirect feedback effects on the temporal organization of behavior and physiology, which can be affected by reproductive events through direct or indirect feedback effects. In humans, changes in sleep disorders and changes in body temperature rhythms have been associated with menstruation, pregnancy, and menopause. In a similar way, during the estrous cycle in rodents, circadian rhythms get altered [12,13,14,15,16], and phase advances of locomotor activity and higher total activity during stages of proestrus and estrus relative to diestrus are observed in female rats [9,17].

### 3.2 Circadian rhythm: Suprachiasmatic nucleus (SCN), Peripheral clocks and *CLOCK* gene

The role of the involvement of circadian rhythm and its circadian *Clock* genes is studied in a multitude cellular functions, biochemical processes, and physiological activities, like basic gut-related phenomena like motility, acid secretion, and digestive enzyme production depends on the circadian rhythm. Maintenance of entire human body functions like respiration, digestion, nervous system, circulation, body temperature, muscular system, cardiovascular system, immune system, hormonal regulation and balancing of reproductive and endocrine system [1,2,6,15,16]. The central clock of the circadian rhythm is articulated via the suprachiasmatic nucleus (SCN) present in the hypothalamus region of the brain, where the transformation of light signals entered into the retina occurs. These biochemical signals transpire into the respective peripheral clocks. These peripheral clocks are dispensed throughout the body parts, in almost every tissue type. The reproductive system is maintained by the hypothalamic-pituitary-gonadal (HPG) axis, controlled by the bidirectional gene expression between the central circadian clock, which is situated in the SCN and the peripheral circadian clock, in the ovarian and uterus tissue (peripheral organs) in the mammalian female reproductive system. Even though the peripheral circadian clock works with an autonomous signalling mechanism using *Clock* genes to sustain its own rhythmic cycle, the entire HPG axis is synchronized by the SCN. This synchronization of peripheral circadian clock occurs with the help of *Clock* genes like circadian locomotor output cycles kaput (*CLOCK*), brain and muscle ARNT-like 1 (*BMAL1*), cryptochrome (*CRY 1/2*), and period (*PER 1/2/3*). These clock gene expression in peripheral organs and in the peripheral circadian clock were highly regulated by the SCN signalling, hence acting as a central circadian clock or molecular clock. In most of the female reproductive organs, like the uterus, fallopian tube, ovaries, cervix, vulva, etc., the *Clock* genes like *CLOCK, BMAL1, CRY ½* and *PER 1/2/3*, are expressed and help locally in controlling the peripheral circadian clock [1,2,9,15,16,18,19,20,21,22]. By this regulatory activity of *Clock* genes, the hormonal balance is maintained, hence influencing the most important processes like ovulation, menstruation, fertility, uterine function, implantation, pregnancy and fetal development. Further these *Clock* genes, regulate the nuclear receptors and vice versa. The *Clock* genes like *CLOCK* and *BMAL1* influence the hormonal and metabolic pathways by having a direct regulation on the nuclear receptors like, nuclear receptor subfamily 1, group D, member 1 (NR1D1/REV-ERB), REV-ERBα, and retinoid-related orphan receptor alpha (RORα), which act as feedback loop regulators. Here, in the bidirectional regulation, the *BMAL1* gene expression is being activated by *RORα* and repressed by *REV-ERBα*. Also, some nuclear receptors like androgen receptors, estrogen receptors, progesterone receptors and glucocorticoid receptors, bind directly to the promoter region of the *Clock* genes. Multiple metabolic and hormonal pathways were regulated by PPAR receptors. Based on a transcription-translation feedback loop featuring the transcription factors *CLOCK*, *BMAL1*, *CRY*, and *PER*, the mammalian clock functions [18,19,20,21,22]. Forming a heterodimer, *CLOCK* and *BMAL1* drive the transcription of target genes, including those genes encoding their own repressors, period (*PER1, PER2, PER3*), and cryptochrome (*CRY1, CRY2*) are the target genes for *CLOCK* and *BMAL1*, as they drive the transcription of these genes. Repression of *CLOCK* and *BMAL1* occurs by dimerization of *PER* and *CRY*, allowing the cycle to repeat. Transcription of approximately 43% of all protein-coding genes is regulated directly or indirectly by this core *CLOCK* setup. By this bidirectional regulation of nuclear receptors, the circadian rhythm in the female reproductive system is well balanced [19,20,21,22].

### 3.3 Circadian rhythm: nuclear receptors

Nuclear receptors (NRs) are regulated by *PER, CLOCK*, and *CRY* genes by a variety of mechanisms [23,24,25,26], and coordinated pathways such as lipid, glucose, and xenobiotic metabolism are linked with time of day information via the rhythmic abundance of endogenous ligands and the rhythmic transcription of coactivators and corepressors [21]. When a xenobiotic ligand binds to the NRs, pregnane X receptor (*PXR*), and constitutive androstane receptor (*CAR*), it induces the expression of proteins required for xenobiotic detoxification. NRs are tightly associated with the circadian clock and its regulation of the 24-hour day/night cycle. Many NRs are rhythmically expressed, and two such NRs are *REV-ERBα/β* and *RORα/γ* being the main players in core *CLOCK* function [21,22]. *CRYs* and NRs co-occupy many genomic sites, as evidenced by the analysis of genome-wide DNA binding. NRs co-repressors *NCOR1*, *SMRT*, and *CRYs* share many markable features biochemically, they do not require a CoRNR box to interact with NRs [27]. Except at the C-terminal tail, *CRYs* are compact in structure [28,29]. Michael et al., (2017) reported that NRs interact directly with the secondary pocket of *CRY1*, suggesting that the *CRY1* area may serve as an essential site of interaction with the transcription factors involved in CRY-mediated repression.

### 3.4 Circadian rhythm: nuclear receptors and *CRY* gene

The *CRY* gene repression of *PXR* or glucocorticoid receptor (GR) is weak when compared with the *CRY* repression of *BMAL1: CLOCK* genes. It is stipulated that a distinct mechanism may be involved in the repression of NRs by *CRYs*, which is yet incomprehensible [9,30]. The *CRY* gene, *BMAL1* gene, and *CLOCK* gene were managed by numerous NRs like farnesoid-X-receptor α (*FXRα*), liver X receptor α (*LXRα*), retinoid X receptor α (*RXRα*), pregnane X receptor (*PXR*), small heterodimer partner (*SHP*)/nuclear receptor subfamily 0, group B, member 2 (*NR0B2*), liver receptor homolog-1 (*LRH-1*), peroxisome proliferator-activated receptor ligands (*PPARα, PPARβ, PPARγ*), glucocorticoids, retinoic acid, thyroid hormone receptor, estrogen receptor, androgen receptor, mineralocorticoid receptor, progesterone receptor, RAR-related orphan receptor (*RORα, RORβ, RORγ*), and several transcripts of NRs were modulated by *BMAL1* and *CLOCK* [31,32]. The ligands that activate the NRs comprise a variety of endogenous hormones, vitamin D, and xenobiotic endocrine disruptors that are lipophilic in nature. In female reproductive system the peripheral circadian clock is mainly perpetuated by multitudinous NRs based pathways and participation of NRs like estrogen receptor, progesterone receptor, androgen receptor and *PPAR* receptors in influencing fundamental gene expression.

### 3.5 Circadian rhythm: nuclear receptors and estrogen

NRs were broached as orphan receptors due to their unclear endogenous ligand binding. Few orphan receptors like PPAR (peroxisome proliferator-activated receptors) and LXR (liver X receptor) bind to metabolic intermediates like sterols, fatty acids, and bile acids. Among these nuclear receptors, PPARγ, estrogen receptors, especially ERα and ERβ, progesterone receptors (PR-A and PR-B), and androgen receptors have an associated role in maintaining the uterus and other female reproductive organs [31,33,34,35]. The estrogens exert their effects and actions through their interaction with specific estrogen receptors (ERs), namely ERα and ERβ [36]. They are associated with the family of ligand-regulated transcription factor of the steroid nuclear receptor superfamily and control gene transcription through the estrogen responsive element (ERE). When ERβ is coexpressed with ERα, it suppresses, inhibits, and counteracts ERα-mediated gene expression [37]. The gene that codes for ERα (ESR1) is being managed by seven different promoters, which generate to different transcripts [38]. The coactivators of ERα have a common LXXLL motif that binds to the AF-2 domain or ligand binding domain (LBD) to regulate transcriptional activation, ERs form dimers [39]. The ERα is predominantly expressed in the hypothalamus, endometrium and ovarian stromal cells [40]. While ERβ is primarily expressed in the ovarian granulosa cells (mostly in primary, secondary, and mature follicles), heart, lungs, bone, uterus [41], intestinal mucosa, glandular epithelial cells, prostate, and endothelial cells [42]. ERβ assists in the regulation of cell fate in the human endometrium, and almost all endometrial cell types express ERβ, and abnormality in ERβ expression is observed in all benign and malignant endometrial proliferative diseases [41]. Anti-proliferative effects were observed in ERβ and therefore antagonize the actions of ER-α in reproductive tissue [43]. ERα is the prime signalling mediator of estrogens in a wide range of organs and tissues, which instigate pleiotropic effects [44]. The eNOS expression in secretory endometrium is being modulated, especially in epithelial glands, when estrogen interconnects with ERα or ERβ, and some estrogen-related receptors (ERRs) upregulates eNOS mRNA and protein expression activity in early secretory endometrial glands [45]. The paracrine and autocrine mechanisms of proliferation and differentiation in uterine endometrial tissues are in response to estrogens. The proliferation of uterine endothelium requires the presence of functional ER-α in the underlying stroma but not the epithelium, and vice versa in the differentiation of the uterine epithelium, requires functional ERα in the epithelium [46]. ERα synchronizes the uterine and vaginal epithelial cell proliferation in mouse, stimulated by estrogen, whereas ERβ inhibits cell proliferation. Estrogen contributes to altering circadian rhythms, as evidenced in a study where an increase in both the amplitude and bout length shortened the period of locomotor activity of female rats and hamsters as an aftereffect of estrogen implants [9,12,13,14,47]. The presence of progesterone hinders estrogenic activity, suggesting that complex regulation of the hormonal environment throughout the estrous cycle is mandatory for the observed behavioral effects. The genes and the protein products in the circadian clock are organized into interlocking positive and negative transcriptional and translational feedback loops that regulate circadian rhythm generation in the SCN, and estradiol escalates the activation of SCN neurons and tissues [9,48]. An important study exhibited that the similar molecular feedback loops found in SCN cells, are found in a majority of peripheral organs like the ovary, uterus, pancreas, intestine, liver, adipocytes, skeletal muscle, endocrine system, cardiovascular system, immune system, metabolic system, and reproductive system [49]. Evidence also supports the fact that many of these peripheral clocks can continue to oscillate even in the absence of the SCN [50]. There are probable chances of peripheral oscillators locally regulating physiology, though they receive modulatory timing cues from the SCN. Even though many studies have explained the expression of circadian clock genes in the ovary [51,52] and uterus [53,54], the physiological function of *Clock* genes in tissues of the female reproductive system remains a lacuna [55,56]. The hormone estrogen, which is generally responsible for producing an anti-obesity and anti-diabetic effect in females through the suppression of food intake, has been found to be critical in balancing the expression of various circadian clock genes [9,47]. Also, genetically modified transgenic mice, which lack a functional aromatase gene, have reduced levels of estrogen and develop obesity [57], effect of low estrogen levels on escalated obesity has been linked to ERα [58]. A recent study observed that during wheel running activity, high levels of estrogen induce an early onset of locomotor activity with a greater amplitude and overall bout length, specifically on the evening of proestrus. Despite the physiological functions of the gene remaining unclear, reports have surfaced of reproductive dysfunction in mice that possess *Clock* gene mutations [59,60]. A female *Clock* mutant mice, which carried a 51-amino acid deletion in the transcriptional activation domain of the CLOCK protein, failed to show circadian rhythm expression or even regular estrous cycles. These mice also failed to produce a surge of LH on the day of proestrus [19]. In a knockout study, *αERKO* female mice were found to be infertile since there is a hypoplastic uteri and hyperemic ovaries with no corpora lutea due to a lack of negative feedback stimulation of LH by estrogens [61]. The mRNAs of both ERα and ERβ are co-expressed in rat corpus luteum during pregnancy, and the mRNA expression is up-regulated by placental lactogen and prolactin [62,63]. Also, in mice, knockout of the *BMAL1* gene resulted in a disrupted HPG axis, damaged gametes, weakened reproductive organs, and impeded steroidogenesis [64]. An observable link is seen where estrogen differentially controls the expression of the *PER1* gene and the *PER2* gene, along with the central and peripheral molecular clocks of the body, as well as between both the reproductive and non-reproductive tissues of the female mammal, particularly in the uterus, with the help of an estrogen receptor-mediated response [19,54]. Researchers have distinguished half-ERE sequences arrangements in the mouse *PER1* promoter [65]. Rats with ovariectomies treated with chronic estradiol showed no change in the *PER1* expression rhythm in the SCN and cortex, while the *PER1* expression rhythm in the liver and kidney appeared to escalate in amplitude and a delay in phase [65]. *PER1* expression in the uterus and uterine stromal cells was likewise elevated in rats after ovariectomies after receiving estradiol treatment [66]. The mechanism by which estrogen controls *PER1* expression is ambiguous; it’s uncertain whether estrogen exerts its regulation directly through half-ERE sites or if it acts indirectly. In human mammary epithelial cell lines with circadian clocks, the human Esr1 (ERα) gene exhibits circadian rhythmic expression despite having an incomplete E-box [67,68]. On the other hand, human breast cancer cell lines that are lacking the typical conventional circadian rhythm genes show non-rhythmic expression of ERα [67]. Furthermore, in MCF-7 cells, an additional luciferase reporter’s activation was diminished by a mutation in the E-box in the ERα estrogen response element (ERE) [68]. These findings imply that CLOCK:BMAL1 controls ERα expression through its E-box; however, an experimental test of this direct regulation is lacking. When the mouse lung is kept in complete darkness, the expression of ERβ mRNA follows a circadian pattern [69]. In the skeletal muscle of mice, the mRNA of ERβ, moreover, exhibits circadian rhythm, which is vanished or eliminated in *BMAL1* knockout animals [69]. Further in vitro research demonstrated that CLOCK/BMAL1 regulation of ERβ transcription was suppressed by altering the E-box in the mouse ERβ promoter [68,69]. All these research investigations and studies reveal that, in mice, the circadian rhythm controls directly by regulating ERβ expression through its E-box sequence in its gene. From the above evidence, it is clear that ER receptor subtypes directly regulate *PER1* expression and indirectly synchronize *Clock* and *BMAL1* in the uterus and ovaries. Endometriosis is an estrogen-dependent disorder, characterized by elevated levels of ERβ relative to ERα. This is linked to suppressed or reduced activity of the progesterone receptor and intensified levels of cyclooxygenase-2 (COX-2), which contribute to inflammation and progesterone resistance in endometriotic stromal cells [68,69,70]. Neonatal expression of ERβ is predominant for the inhibition of epithelial cell proliferation and accumulation of p27 protein in the mouse uterus, and suppression of ERβ function in the uterine epithelium during the neonatal stage could be linked to an elevated risk of proliferative disease in adulthood [71]. Estrogen implementation modulates the expression of the *Clock* genes in SCN, reproductive, and non-reproductive tissues in-vivo, according to another study that tested a hypothesis and rightfully concluded that the hormone can directly regulate the *Clock* gene expression in the uterus [9,69,72].

### 3.6 Circadian rhythm: nuclear receptors and progesterone

Progesterone receptors are expressed in innumerable compartments of the uterus, in the endometrial (internal epithelial layer) and myometrial regions, and in the stroma and their spatiotemporal expression is regulated by both estrogen and progesterone [73]. The PR gene promoter region has clusters of estrogen response element (ERE), which are indispensable for activation of the PR gene by the ligand bound estrogen receptors (ERs) [74]. Hence, consequently PR is regarded as the marker for estrogen action [75]. The PR were acted upon by progesterone, to impede the proliferative response of the epithelium to estradiol, and instigate proliferation of the underlying stroma [76]. The secretion of progesterone after ovulation inhibits the proliferative activity of endometrium and persuades the complex secretory activity. This process initiates with the transportation and polarization of glycogen to the cell’s apical region through the microfilaments. The Golgi apparatus packs the glycogen to form secretory granules, which are expelled out into the glandular lumen. These secretory changes take place only in an estrogen-primed endometrial layer [77]. PR-B are anti-inflammatory in action; hence, during pregnancy, the uteri epithelial endometrial cells manifest elevated levels of PR-B, promoting quiescence and during parturition. And the labor stimulated by the proinflammatory gene expression of PR-A by inhibiting PR-B action of anti-inflammatory activity. The discrete inhibitory activity of hPR-A, which is masked in the context of hPR-B, confesses that these two receptor isoforms may interact with divergent proteins of transcription factors, co-activators, and co-repressors within the cell [78,79,80].

In direct contrast to estrogen, progesterone was seen to reduce locomotor activity but not the rhythmic cycle. We know that circadian rhythm generated by the master pacemaker SCN works in transcriptional and translational feedback loops, which results in the coordinated functioning of all circadian rhythmic activities [19,51,52,53,54,81]. However, an individual study has discovered that the uterine hormone progesterone, was seen to reduce locomotor activity in general, without affecting the overall period of rhythmic activity. Due to the uterus being composed of a variety of heterogeneous cell types with different functions, ovarian steroids have been shown to regulate the proliferation as well as differentiation of the endometrial stromal cells [81,82]. The cell type-dependent reciprocation of progesterone and the forkhead box class O family of transcription factors (FOXO1A), associate with one another in the human endometrial cell and balance a secretory by-product of the endometrium decidua called insulin-like growth factor-binding protein-1 (IGFBP1) [83]. The crosstalk interaction between STAT3 and PR signalling is being essential for ensuring successful implantation [79]. In rodent uterine epithelial cells, the expression of progesterone receptor (PR) is independent of estrogen and ERα, but the transcription factors that regulate the PR gene are dependent on estrogen and ERα [84]. Also, during endometrial decidualization (especially during the transition of stromal proliferation to stromal differentiation) in human females, the *PER1* gene expression escalated in direct proportion to the PR. The instantaneous binding of PR to the promoter region of the *PER1* gene initiates the transcription activity of the gene [85]. An isoform-specific PR ablation study showed that PR-A absence conciliates progesterone signalling in the reproductive tract [86,87], but PR-B signalling is a necessitate for progesterone signalling in the mammary gland [80,87]. TNF-α may modulate the withdrawal of progesterone in human decidua after the labor onset [88,89]. Hence, for successful preparation of uterine epithelium for receptivity, endometrial stromal cell decidualization, implantation, and directly modifying the circadian clock of the reproductive axis at every level, progesterone receptor (PR) signalling is required in both humans and rodents [75,86].

### 3.7 Circadian rhythm: nuclear receptors and androgen

The androgen receptor (ARs), belongs to nuclear receptor subfamily 3, group C, member 4 (NR3C4), is a type of nuclear receptor, which is activated by the androgenic hormones like testosterone. These androgen receptors are predominantly localized in the human granulosa cells, theca cells, and stromal cells [90,91,92]. Past studies point to the fact that androgenic effects on circadian responses can be directly attributed to the SCN and extra SCN sites, where the overall quantity and amplitude of the activity may not have a base in the master circadian clock. Even though the site of androgenic hormone response remains rhythmically delineated, it is believed that specific behavioral responses, like the period and precision of the activity, are controlled by the circadian pacemaker directly. Androgen receptors play an important role in estrogen induced uterine epithelial cell proliferation, which improves and nurtures female fertility and acts via intra-ovarian and neuroendocrine signalling mechanisms to regulate ovarian folliculogenesis, ovulation, endometrium cycling, and proliferation [91,92,93,94,95]. AR signalling pertains to the decidual cell differentiation from human endometrial stromal cells [96]. AR induces IGF-I and then amplifies the ERα signal to induce epithelial cell proliferation in a paracrine fashion in the stromal cells, by which androgens exert an uterotrophic effect in the rat uterus [97]. A knockout study in mice has shown that AR is extremely essential for normal female fertility [98]. Therefore, concluding that these activities of the circadian rhythm are rightfully regulated by androgens [92,93,95].

### 3.8 Circadian rhythm: nuclear receptors and PPAR

The peroxisomes are microbodies that are existent abundantly in mammalian liver and kidney [99]. The membrane of peroxisomes is not rich in enzymes, but the matrix is rich in enzymes, which mostly comprise catalases and peroxidases. Most familiar enzymes of matrix are cytochrome b5, NADH- cytochrome b5 reductase, which forms the integral proteins of endoplasmic reticulum [100]. These peroxisomes are involved in the metabolism of branched chain fatty acids, very long chain fatty acids, polyamines and D-amino acids, synthesizing hydrogen peroxide through β-oxidation [101]. The PPARs are a cluster of nuclear receptor proteins that are expressed in liver, kidney, adipose tissue, colon, heart, and muscle [102].

Technical advances in PPAR biology are one of the emerging fields of study in metabolism and its relationship to homeostasis with the help of circadian regulated factors, placing PPARγ in the center of yet another crucial regulatory pathway. PPARγ functions as a transcription factor in the synchronization of gene expressions, which are very crucial in the modulation and regulation of metabolism of protein, carbohydrate, and lipid, cellular differentiation, and development of organisms [103]. PPARγ and the retinoid X receptor (RXR) combined together to form a heterodimer compound, which subsequently binds to particular or specific distinct regions of the target genes of the DNA [104]. Natural ligands such as polyunsaturated fatty acids (PUFA), metabolites of prostaglandin, and some of the synthetic ligands like thiazolidinediones (TZDs), also referred to as glitazones/rosiglitazone/pioglitazone/troglitazone can activate PPARγ [105]. PPARγ has a vital role in various biological processes such as glucose homeostasis, adipogenesis, atherogenesis, and inflammation. The fat mass level and cell proliferation control were modulated, adjusted and regulated by PPARγ [106].

The inner-cell mass (ICM) of the blastocyst stage and trophectoderm layer constructed after the gastrulation possesses an elevated level of PPARγ expression [107]. After ovulation, the expression of PPARγ in the corpus luteum becomes exorbitant and slowly regresses, if fertilization or embryo implantation does not, and also for the appendage of the embryo to the endometrium and for proper functional development of the placenta, PPARγ plays the multitudinous crucial role [108]. During the LH surge in granulosa cells, the PPARγ expression is downregulated [109]. PPARγ regulates and modulates many proteases like matrix metalloprotease-9, plasminogen activator, and plasminogen activator inhibitor, which are requisite for follicular rupture and tissue remodeling during folliculogenesis and corpus luteum formation [110,111]. Lutein cells secrete vascular endothelial growth factor (VEGF), which assists in maintaining the corpus luteum function during pregnancy, and PPARγ balances and regulates the function of VEGF and promotes angiogenesis [112]. PPARγ upregulates VEGF in human vascular smooth muscle cells to control and regulate the function of the corpus luteum to secrete progesterone, which is involved in implantation and gestation [113]. PPARγ expression is detected in gestational tissues like amnion, choriodecidua, and placenta [114]. Deletion of PPARγ in a tissue-specific manner in the ovary, lymphocytes, and epithelial cells in mice leads to steer in minimizing the rate of fertility, causing an altered ovarian folliculogenesis and a decreased synthesis of progesterone from the corpus luteum. This results in an unfavorable embryo implantation window [115]. Inactivation of PPARγ leads to impaired placental vascularization, and results in the death of the embryo [116]. In an *RXRα-/-* knockout study in mice, there were difficulties in placental development, stating that *PPARγ-RXRα* heterodimer formation is most essential for placentation [117]. The prostaglandin D2 (PGD2) is a major and significant prostaglandin signalling molecule that is prominently expressed during pregnancy. The uterine contraction during parturition involves the action of prostaglandin. This PGD2 acts as a natural ligand for PPARγ [118]. Also, hydroxyeicosatetraenoic acids, a derivative of arachidonic acid [119], act as ligands of PPARγ, and are produced in uterus epithelial cells during implantation, and inhibition of this ligand has lowered and diminished the implantation rate [120]. Cyclooxygenase-2 (COX-2) catalyzes the prostaglandins produced by endometrium, myometrium, and fetal membranes during parturition [121] and later PPARγ promotes downregulation of COX-2, stimulating progesterone secretion and reducing endometrial inflammation after parturition and thus maintaining and conserving the quiescence of the uterus [122]. And in endometriosis, PPARγ activation suppresses the biosynthesis of estrogen and prostaglandin D2 (PGD2) signalling and thereby inhibits the growth and survival of human endometriotic cells (endometriosis) in murine and baboon models [123].

Observations from a specific study find that people working night-shifts with disturbed circadian rhythms are more prone to the induction of suffering from cardiovascular complications, disrupted reproductive cycles, sleep disorders, and metabolic syndrome. In this paper, it was concluded that circadian gene mutations are associated with sleep disorders while at the same time affecting the overall body composition of the individual [124,125,126,127]. Tight evidence can be found for the strong connection between the PPAR family, the circadian network, and their metabolic outputs. Whereas another gene, PPARα controls the *BMAL1* gene transcription expression, and PPARy is involved in the overall metabolic regulatory pathway, along with supervising the rhythm of *Per2-luciferase*. New discoveries that show the multifunctional nature of PPARy subsequently open doors for the development of highly targeted novel agents on adipocytes and uterus progenitors with minimal to no side effects on the skeleton [124,125,128,129].

### 3.9 Circadian rhythm: Ovary -an important peripheral clock

In the ovaries of rats, mice, and bovines, the various clock genes were shown to oscillate [130]. Ovary is one of the most predominant peripheral clocks. The expression of the *CLOCK* gene in the ovaries of rats, mice, and ruminants, has been described by multiple experimental studies. *CLOCK* gene expression was localized in granulosa (GC), thecal (TC), and luteal (LC) cells of the rat ovary by the in-situ method. These central clock in ovaries are regulated by various mechanisms involving nuclear receptors to control reproductive peripheral clocks. The *BMAL1* and *CLOCK* heterodimerize interacting with the E-Boxes in the promoters of Clock-controlled genes, leading to positive transcription of the TTO loop. The transcription of *PER* and *CRY* homologs is initiated by these transcription factors. *PER* and *CRY* can inhibit the activity of *CLOCK* and *BMAL1*, therefore, the TTO loop’s transcription is repressed. The other accessory proteins that modulate the loop include, D-box binding protein (DBP) and the nuclear orphan receptors like ROR and NR1D1/REV-ERB, which modulate the activity of the loop [1,2,131,132]. The phosphorylation of period proteins involves Casein kinases 1 and 2 (CK1 and CK2). It was reported that the delay in circadian phase was due to CK1 inhibition. The nuclear localization signal (NLS) of *PER1* was concealed by the CK1ɛ phosphorylation; in turn, it results in cytoplasmic localization in HEK293 cells. *PER1* interacts with PP1 (Protein phosphatase 1), and PP1 dephosphorylates *PER1* and aids in the cytoplasmic localization of *PER1*. It is speculated that the balance between CK1δ/ɛ phosphorylation and dephosphorylation by PP1 might be a key regulator of the post-translational regulation of the period proteins [131,132]. At early night, *PER1* and *PER2* mRNA expression peaked, rhythmic independent of the phase of the estrous cycle and consistent when rats were subjected to constant darkness [132]. *PPARα* is one of the nuclear receptors, and it is well established that its expression is found in the rat’s ovary, working based on the heterodimerization with RXR. Circadian expression of PPARα is regulated directly by CLOCK protein via the E-box-rich region in the second intron in-vivo and in-vitro, and *CLOCK* plays an important role in lipid metabolism by regulating the circadian transactivation of *PPARα* in the mice [132,133]. As per a study, there was circadian variation of *PER1* and *PER2* expression in rat ovaries, the time-dependent intracellular localization of *PER1* and *PER2* was demonstrated by immunofluorescence [134]. Luteinizing hormone secretion, ovulation, and estrous cycling in rats were evidenced when there were diminished SCN lesions [130]. BMAL1-KO male and female mice were infertile due to decreased sex steroid hormone production, delayed estrous cycle, and implantation defects [135,136]. Although extensive research has been carried out on the reproductive peripheral clock (ovary) and PPARα activation by the E-box in the CLOCK protein, there is no evidence directly linking this kind of activation by the E-box in ovary. Hence, further research is warranted to find out the exact mechanism of regulation of *CLOCK* genes in the peripheral clocks [1,130,131,133,136].

### 3.10 Circadian rhythm: conclusion

To conclude the fact that, overall, most of the SCN and NRs-mediated circadian rhythm axis of most of the peripheral and reproductive tissues are subsequently and ultimately responsible for maintaining the unique sexual dimorphism that is observed in females until the decline, which is initiated after the postmenopausal period [137]. Fertility in women is determined by the pertinacious and precise timing of hormone release patterns, which are regulated by the HPG axis. The SCN and NRs cooperatively modulate the circadian rhythms, genes of reproduction function, and locomotor activity. The circadian clocks orchestrate the environmental and physiological signals, resulting in cell’s endogenous rhythms controlled by the molecular clocks (genes – *CLOCK, BMAL1, PER, CRY*). Any imbalance in the circadian rhythm will affect, transform, and damage the reproductive axis by modifying the hormone release patterns and, therefore, altering the expression of nuclear (steroid) hormone receptors in the female reproductive system [138]. On the other hand, hormones and steroids incorporated in the regulation of female reproductive functions are linked to the circadian timing system. Therefore, the consequences and impact of irregular circadian patterns on the reproductive system and the molecular mechanisms involved in them need to be addressed in the future [138,139,140]. Even though it is perceptible that ERα, ERβ and PR’s expression is observed in SCN neurons and tissues [137], the reciprocal and bidirectional synchronization among the SCN tissues and NRs in regulating the HPG axis of female reproductive system signalling still requires a better interpretation [19,30,138,139,140].
